# The complete mitochondrial genome of *Orthonychiurus folsomi* (Collembola: Onychiuridae)

**DOI:** 10.1080/23802359.2019.1703581

**Published:** 2019-12-18

**Authors:** Haifeng Yao, Zhijing Xie, Jie Dong, Xin Sun

**Affiliations:** a Northeast Institute of Geography and Agroecology, Chinese Academy of Sciences, Changchun, China;; b University of Chinese Academy of Sciences, Beijing, China;; c J.F. Blumenbach Institute of Zoology and Anthropology, University of Göttingen, Göttingen, Germany;; d College of Plant Protection, Nanjing Agricultural University, Nanjing, China

**Keywords:** Mitogenome, gene order, widespread species, phylogeny, springtail

## Abstract

The complete mitochondrial genome of *Orthonychiurus folsomi* (Schäffer 1900) was sequenced, assembled, and annotated. The mitochondrial genome of *O. folsomi* has a length of 15,283bp and comprises 13 protein-coding genes, 22 tRNA genes, and two rRNA genes. Two tRNA genes *trnS(uga)* and *trnQ* have changed position. A phylogenetic tree of Onychiuridae species showed the polyphyly of this family.

Collembola is one of the most widespread and abundant soil animals in terrestrial ecosystems and plays an important role in soil nutrient cycling and plant litter decomposition (Rusek 1998; Filser 2002). About 9000 species have been reported worldwide so far (Bellinger et al. 1996–2019). In contrast, only 16 species of mitogenome sequences are available in public databases and the evolutionary relationships of Collembola are still controversial (Leo et al. 2019; Wu and Chen 2019). The family Onychiuridae is mostly euedaphic in different ecosystems, and some species are crop pests leading to reduce yields and productivity (Baker and Dunning 1975; Joseph et al. 2015). With only three species of mitochondrial genomes recorded (Leo et al. 2019), the phylogenetic relationship of the groups of Onychiuridae is far to be resolved. Here we performed the sequencing, assembly, and annotation of the mitochondrial genome of *Orthonychiurus folsomi* (Schäffer 1900), and described its molecular characteristics.

Specimens of *O. folsomi* were collected from the litter in Zijinshan Park, Nanjing, China (32.076°N, 118.860°E, NCBI BioSample accession SAMN11656301) on August 15, 2016. The specimen (specimen Accession number 0516OF) and its DNA were deposited in the Nanjing Agricultural University, Nanjing, China. Genomic DNA was extracted using the Qiamp DNA Micro kit (QIAGEN GmbH, Shanghai, China). DNA concentration was measured by Qubit 3.0 using Q33230 Qubit™ 1X dsDNA HS Assay Kit. Genomic DNA was sequenced on HiSeq X Ten platform (Tianjin Novogene Bioinformatics Technology Co., Ltd, China). The *cox1* was used as a seed sequence for the mitochondrial genome assembly. The mitogenome was assembled with NOVOPlasty 2.6.6 (Dierckxsens et al. 2017), annotated with mitoZ and MITOS2 WebServer (Yokobori and Pääbo 1995; Lupi et al. 2010), and deposited in Genbank (accession number: MN661001).

The complete mitochondrial genome of *O. folsomi* is a closed circular molecule of 15,283 bp and contains the set of 37 genes usually found in metazoans, including 13 protein-coding genes, 22 tRNA genes, two rRNA genes. The length of gene varies from 52 bp (*trnC*) to 1729 bp (*nad5*). The nucleotide composition is 35.41% adenine, 36.79% thymine, 10.06% guanine, and 17.74% cytosine, biased toward a high A + T content (72.20%), as typically found in hexapod mtDNAs. A non-coding region, A + T-rich region, located at the gene junctions *trnQ*/*trnI* (809 bp) was detected. The gene order is identical to the known Onychiuridae species with the translocation of *trnQ* and *trnS*(*uga*), compared to the common mitochondrial genome order of Collembola (Carapelli et al. 2014).

The 13 protein-coding genes (PCGs) were aligned using MAFFT v.7.394 (Kazutaka and Standley 2013), trimmed using trimAL v.1.4 (Capella-Gutiérrez et al. 2009), and concatenated using FASconCAT-G v1.04 (Kück and Longo 2014). A phylogenetic tree was constructed based on nucleotide sequences of 13 PCGs in IQ-TREE v1.6.3 (Nguyen et al. 2015) using the GHOST model with ultrafast bootstraps and SH-aLRT test (UFBoot, Hoang et al. 2018; Guindon et al. 2010). The species *O. folsomi* clustered with *Thalassaphorura orientalis* (Stach 1964), but *Thalassaphorura encarpata* (Denis 1931) clustered with the species of the subfamily Tetrodontophorinae (Figure 1). The results show that the monophyly of the subfamily Onychiurinae and the genus *Thalassaphoura* is not supported.

**Figure 1.**
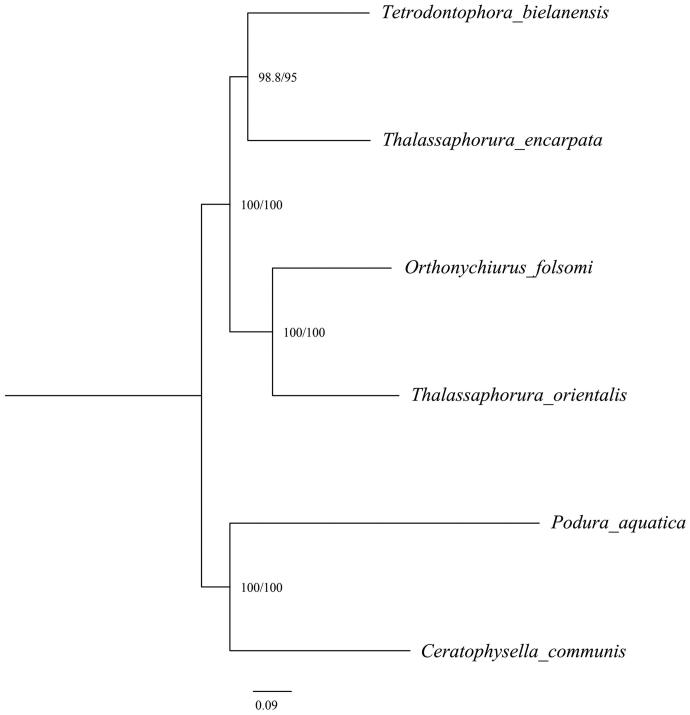
Maximum Likelihood phylogenetic tree of *Orthonychiurus folsomi* and five other species inferred from nucleotide sequences of 13 PCGs under GHOST model. Bootstrap support values are displayed at the nodes. The following species are used to construct phylogenetic trees: *Orthonychiurus folsomi* MN_661001 (Collembola, Onychiuridae), *Tetrodontophora bielanensis* NC_002735 (Collembola, Onychiuridae), *Thalassaphorura encarpata* MK_423968 (Collembola, Onychiuridae), *Thalassaphorura orientalis* NC_006074 (Collembola, Onychiuridae), *Podura aquatic* NC_006075 (Collembola, Poduridae), and *Ceratophysella communis* MK_409686 (Collembola, Hypogastruridae).
